# N-of-1 Healthcare: Challenges and Prospects for the Future of Personalized Medicine

**DOI:** 10.3389/fdgth.2022.830656

**Published:** 2022-02-11

**Authors:** Kui You, Peter Wang, Dean Ho

**Affiliations:** ^1^The Institute for Digital Medicine (WisDM), Yong Loo Lin School of Medicine, National University of Singapore, Singapore, Singapore; ^2^The N.1 Institute for Health (N.1), National University of Singapore, Singapore, Singapore; ^3^Department of Biomedical Engineering, Faculty of Engineering, National University of Singapore, Singapore, Singapore; ^4^Department of Pharmacology, Yong Loo Lin School of Medicine, National University of Singapore, Singapore, Singapore

**Keywords:** digital health, digital medicine, personalized medicine, N-of-1, artificial intelligence

## Introduction

Personalized medicine has the potential to drive practice-changing medicine for indications ranging from oncology to transplantation and beyond. Conventionally, the field of medicine has been harnessing population-based approaches to address the broad spectrum of intervention and diagnosis and domains that span drug development to drug dose recommendations (1, 2). For example, drug development traditionally follows an established workflow from drug discovery to clinical trials followed by approvals and subsequent commercialization (3, 4) which can be time-consuming and costly. Of note, therapeutics resulting from successful trials are usually administered in a population-based and one-size-fits-all fashion, as regimen administration guidelines are determined *via* dose expansion or maximum tolerated dose (MTD) (5–7). Population-determined protocols can generally pinpoint suitable treatments/interventions for individuals, but the emergence of artificial intelligence (AI) and digital medicine offers the potential to truly optimize patient outcomes (8). Optimization, in this context, is also a longitudinal process that accounts for dynamically variable outcomes whereas population-based guidelines conventionally do not account for inter- and intra-patient variability (9). Therefore, personalized medicine/N-of-1 treatment approaches using a patient's own data may be deployed to identify the most suitable interventions on a patient-specific level in order to enhance and sustain treatment with truly optimized outcomes (5, 9–12). However, challenges and questions still need be addressed before integrating personalized medicine into current clinical/treatment guidelines.

## Three Grand Challenges in Personalized Medicine

### Personalized Medicine Using Only a Patient's Own Data

Truly personalized medicine differs from traditional medicine, which relies on population-derived guidelines. While personalized medicine can be defined differently depending on factors that include but are not limited to clinical indications, or intervention and diagnostic employed, among others, we will define personalized medicine as N-of-1 medicine, or the use of only a patient's own clinical data to pinpoint and identify suitable interventions only for that specific patient to both identify individualized regimens and sustain optimized dosing using longitudinal diagnostic platforms. On an individual level, personalized medicine strategies utilize a wide array of clinical information to achieve the optimal clinical outcomes. Current personalized strategies mostly acquire clinical data in two different categories: genomic and phenotypic data. For example, using genomic profiling, clinicians and scientists can determine which drugs the patient may benefit from and ones that may cause severe side effects (13). Moreover, some phenotypic-driven approaches can even realize the optimal treatment strategies using a patient's own phenotypic data, such as reliable biomarker measures corresponding to a quantifiable intervention (e.g., drugs) (5, 12, 14). In a case report, Pantuck et al. harnessed CURATE.AI, an AI-driven optimization platform that used a metastatic prostate cancer patient's prostate-specific antigen (PSA) data to dynamically optimize combination therapy to achieve low PSA values (5). In a similar study, Zarrinpar et al. utilized a phenotypic personalized medicine platform that used only patient-specific data to continuously optimize liver transplant immunosuppression (15). Whether via genetic or phenotypic approaches, N-of-1 medicine in these cases only rely on patient-specific data to optimally dose suitable interventions, instead of population-based medicine that relies on averages across a given population.

Furthermore, cell lines have always been the golden standard in *in vitro* studies for early stage drug development. However, the homogeneity of cell lines may result in a lack of differentiated cell types that represent clinical conditions (16). Thus, cell lines have limited the ability of personalized medicine to identify suitable interventions. The emergence of patient-derived models has overcome some of the limitations of traditional *in vitro/vivo* work (16). Notably, patient-derived xenografts (PDX), which are derived from patient cells/tissues and implanted into immunodeficient/humanized mouse models, have enabled the monitoring and evaluation of various interventions and corresponding responses that more closely represent the original patient's disease condition compared to generalized, cell line-based models (17–19). For example, a patient-derived organoid (PDO) model can utilize patient-specific stem cells to grow organoids that resemble the dynamic behavior of original organs and thus, enable personalized interventions in clinically-relevant contexts (20–22).

### How the Data Is Acquired vs. How Much Data Is Acquired

Realizing truly individualized healthcare will rely on more than technology and the amount of patient data acquired. Putting N-of-1 medicine into practice will also rely heavily on how the data is acquired and the clinical workflows needed to bridge technology ideation with data usage and deployment of next generation personalized medicine platforms.

The spectrum of clinical platforms available for building comprehensive databases include molecular profiling to guide regimen selection (23, 24). These approaches may predict a patient's response to certain treatments and provide personalized regimens. Moreover, PDX samples are often obtained from surgically removed tumors or via biopsies, which are both invasive (25). PDO samples require adult stem cells obtained invasively from blood circulations or bone marrow (26, 27). These strategies have seen increasing prevalence in clinically-relevant settings to actionably identify potential treatment strategies that are targeted to each patient. Importantly, drug dosing and its strong correlation with the composition of drug regimens can be harnessed to even further improve how data pertaining to molecular alteration-driven drug selection can be leveraged to personalize treatment. More specifically, additional data pertaining to dose optimization can be acquired to sustain the practice of N-of-1 medicine in a longitudinal manner.

In multiple prospective clinical studies, CURATE.AI platform was harnessed to dynamically optimize combination therapy for patients using their own clinical data. For instance, an 82-year-old metastatic castration-resistant prostate cancer patient whose treatment was guided by CURATE.AI had blood draw weekly to determine blood serum PSA, which was used to continuously pinpoint drug dosages (5). The platform only required a small number of PSA measurements from corresponding combinations of dosages to initiate the optimization process. Subsequent PSA values were collected to update the optimization platform and to account for the intra-patient variability, or the dynamic changes of the patient's response to treatments (5). Additionally, the quadratic phenotypic optimization platform (QPOP), an AI-driven platform that only requires a minimum amount of experimentally-derived drug combination information, was employed to optimize drug combinations against multiple myeloma (MM) from a pool of 114 drug candidates (28). Without relying on the drugs' mechanism of action, QPOP successfully identified the globally optimal combinations and the respective dosages and subsequently, optimized drug combinations for PDX models and *ex vivo* patient samples to design patient-specific drug combinations against MM (28).

In sum, the frequency and approach for data collection depend on the personalized strategy utilized. However, the approach to access data should carefully consider a patient's quality of life and when possible, non-invasive or minimally-invasive approaches should be utilized. Frequency of data collection varies between strategies, as patient sample-driven regimen development only requires a one-time sample; whereas approaches like CURATE.AI would require longitudinal data collection.

### Can We Harness Digital Platforms to Scale the Deployment of Personalized Medicine?

Digital platforms have already been harnessed to scale the deployment of personalized medicine. In order to achieve better accessibility of personalized medicine, bridging personalized strategies with digital medicine should be prioritized. For example, CURATE.AI can be digitized to enable rapid optimization of patient's treatment outcomes using the patient's own clinical data. The platform has been harnessed to guide prostate cancer combinatorial treatment, and the patient was able to resume normal and active lifestyle (5). On the other hand, CURATE.AI also simultaneously optimized the tacrolimus dosing of multiple post-liver transplant patients, and they were able to be discharged earlier than those under standard of care (15). Digitizing platforms like QPOP can potentially provide more tailored, personalized treatments to patients who are not responding to standard of care (28–30). Therefore, digitizing personalized medicine strategies for deployment in clinical settings is one critical step forward to integrate them into established clinical workflows.

## Conclusion

Truly personalized or N-of-1 medicine represents a new avenue toward practice-changing healthcare. Inter- and intra-patient variability can be addressed by N-of-1 medicine with its dynamic and continuously guided treatments that may achieve better clinical outcomes than those determined from population averages. Despite its advantages, personalized medicine's integration into clinical settings must address the three current grand challenges discussed above, and others along the way. Once these concerns are fully addressed, personalized medicine may be able to fully integrate into clinical settings and provide personalized, optimized, and accessible healthcare to patients.

## Author Contributions

All authors contributed to the article and approved the submitted version.

## Conflict of Interest

DH is a co-inventor of current and pending patents on artificial intelligence-based therapy development. DH is a shareholder of KYAN Therapeutics, which has licensed intellectual property pertaining to AI-based drug development. The remaining authors declare that the research was conducted in the absence of any commercial or financial relationships that could be construed as a potential conflict of interest.

## Publisher's Note

All claims expressed in this article are solely those of the authors and do not necessarily represent those of their affiliated organizations, or those of the publisher, the editors and the reviewers. Any product that may be evaluated in this article, or claim that may be made by its manufacturer, is not guaranteed or endorsed by the publisher.
